# Stable C & N isotopes in 2100 Year-B.P. human bone collagen indicate rare dietary dominance of C4 plants in NE-Italy

**DOI:** 10.1038/srep38817

**Published:** 2016-12-09

**Authors:** Zita Laffranchi, Antonio Delgado Huertas, Sylvia A. Jiménez Brobeil, Arsenio Granados Torres, Jose A. Riquelme Cantal

**Affiliations:** 1 Department of Legal Medicine, Toxicology and Physical Anthropology, Medicine Faculty, University of Granada (UGR), Av. de la Investigación 11, 18016, Granada, Spain; 2 Biogeochemical of Stable Isotopes Laboratory, Andalusian Institute of Earth Sciences (IACT-CSIC-UGR), Av. de las Palmeras 4, 18100, Armilla, Granada, Spain; 3 Department of Geography and Territorial Sciences, Area of Prehistory, University of Cordoba, Plaza del Cardenal Salazar 3, 14071, Cordoba, Spain

## Abstract

C_4_ plants (e.g. maize, millet), part of our current diet, are only endemic of reduced areas in South-Europe due to their need of warm climates. Since the first vestiges of agriculture in Europe remains of C_4_ plants were recorded but their overall proportion in the human diet remains unknown. Therefore, isotopic (δ^13^C and δ^15^N) composition of bone collagen from the skeletal remains (human and animals) of a Celtic population, *Cenomani* Gauls, from Verona (3^rd^ to 1^st^ century BC) in the NE Italy provide a new perspective on this matter. The δ^13^C collagen values of 90 human skeletal individuals range between −20.2‰ and −9.7‰ (V-PDB) with a mean value of −15.3‰. As present day C_4_ plants have δ^13^C values around −11‰, which is equivalent to −9.5‰ for samples of preindustrial age, the less negative δ^13^C values in these individuals indicate a diet dominated by C_4_ plants. This palaeodietary study indicates that some European populations predominantly consumed cultivated C_4_ plants 2100 year B.P. This is supported by the paleobotanical records and ancient Roman sources (e.g. Pliny the Elder), which indicate that millet was a staple food in South-Europe.

The term C_4_ plants refers to the type of photosynthetic pathway (Hatch-Slack or C_4_ cycle) used by these plants, which has less isotopic discrimination than the C_3_ cycle (C_3_ plants). In the C_4_ cycle the first stable compound namely oxaloacetic acid is formed by 4 carbon atoms. This group includes warm climate grasses like maize, millets, sorghum and sugar cane, among others^1^.

Paleobotanical studies have shown the presence of domesticated C_4_ plants (in particular broomcorn or *Panicum Miliaceum* L. and foxtail millet or *Setaria Italica* L. Beauv.) in Central and Eastern Europe as early as 5000-4000 BC for *Panicum Miliaceum* and Late Bronze Age for *Setaria Italica*^2^; while in Asia (China) and in the Caucasian region (Georgia) this is documented as early as ca. 10000 years B.P.^3^,^4^,^5^. Based on the botanical evidence, it seems that millet was first introduced into Central and Eastern Europe from the Steppe regions during the Neolithic period^6^,^7^,^8^. Broomcorn millet seems to have arrived in the Mediterranean zone from the north or the north-east^9^; in northern Italy, millet appears in early Bronze Age settlements (1700-1500 BC)^2^. The earliest records of foxtail millet (*Setaria Italica* L. Beauv.) come from Peigang and Cishan (North China) in the 6^th^ millennium BC^3^. In Europe the first carbonized seeds of foxtail millet appear in the 2^nd^ millennium BC, from Bronze Age settlements in central Europe^10^ and France^11^. It is also reported from the late Bronze age Kastanas site (Macedonia, Greece)^12^. In the Near East the earliest evidence for foxtail millet cultivation dates back to the Iron Age (c. 600 BC) in Tille Höyük, south-east Turkey^7^. However, these data only indicate the presence of millet, but do not reveal their role in the human diet.

C_4_ plants are typical of warm climates and their abundance is highly correlated with climatic factors, such as temperature, precipitation and the degree of aridity. In general, they are not present in environments where night temperatures are lower than 8 °C^13^,^14^. Nowadays in Europe C_4_ biomass is very scarce; however these plants are naturally present in small proportions especially in the SW-Europe (e.g. Spain, Portugal, SW-France and Italy)^15^. Before the onset of agriculture, herbivores collagen isotopic δ^13^C values from S-Italy (32.6 to 13.3 ka B.P.) suggest that C_4_ biomass was practically absent (calculated vegetal biomass is around −25‰ vs V-PDB)^16^. Even during the warmer periods of the Late Miocene, fossil isotopic data indicate the absence of C_4_ biomass in central and southern Europe^17^,^18^. Man has introduced certain species, such as millet, with a planting strategy in the warm season (i.e. summer), possibly as a response to Holocene arid periods^11^.

The stable isotope composition (δ^13^C and δ^15^N) is a very effective tool to study the trophic web in ecosystems^19^,^20^,^21^,^22^,^23^. This methodology is crucial for studying past ecosystems and paleodiets, because direct observation is impossible^24^,^25^,^26^,^27^. In addition this technique allows quantitative analysis, which even in current ecosystems is difficult to obtain with the classical methods of observation. Isotopic carbon traceability suffers less change in the steps of the food chain than isotopic nitrogen one. Therefore, the footprint of primary producers (e.g. plants) is clearer^28^.

The importance of carbon isotopes as a racer for the presence of C_4_ plants in the diet, is that atmospheric CO_2_ is fixed into organic matter by the enzyme PEP carboxylase (C_4_ plants) which discriminates much less against ^13^C than the enzyme RuBisCO (C_3_ plants)^29^. In present day C_3_ plants biomass show typical δ^13^C values around −26‰ (V-PDB), while C_4_ plants are around −11‰ (V-PDB)^30^,^31^, −24.5‰ and −9.5‰ (V-PDB), respectively in preindustrial age, because δ^13^C values of CO_2_ were less negative by 1.5‰ compared to the present values (−8‰ V-PDB). This 15‰ difference between C_3_ and C_4_ plants allows an accurate calculation of the percentage of C_4_ plants in the diet. The δ^13^C_collagen_ values of humans and animals are proportional to the isotopic signal from the base of the food chain (plants), but enriched by 0.8 and 1.3‰ for herbivores and carnivores, respectively^32^. The δ^15^N of the collagen is also related with the food chain and can be used to evaluate the trophic system, because in a particular ecosystem there is a significant enrichment in ^15^N between 3 and 5‰ for each trophic level^25^,^26^,^27^.On the other hand δ^15^N cannot be used to distinguish between C_3_ and C_4_ plants consumption.

There is little knowledge about the nutritional habits of protohistorical populations such as Pre-Roman-Celtic ones. Therefore, we performed an isotopic study to unveil the dietary habits of the Celtic population *Cenomani* Gauls, from the necropolis of *Seminario Vescovile* in Verona (Italy) dated between the 3^rd^ to 1^st^ century BC^33^. This necropolis counts with a minimum of 174 skeletons in a good state of preservation, and the majority of them are non-adults (see Supplementary Information for detailed descriptions of the archaeological context). Surprisingly, we recorded relatively high, i.e. less negatives, δ^13^C_collagen_ values in the human samples, and even in some animals found in the necropolis. In our initial considerations, we suspected the presence of protein in the diet from marine sources, or even anomalous fresh water sources (enriched ^13^C in DIC-Dissolved Inorganic Carbon, “dead carbon” from marine carbonates), or alternatively an extensive consumption of C_4_ plants. The first hypothesis seemed from the beginning improbable, taking into account the geographic location of the necropolis, which was far from the sea (about 120 km). Additionally, the less negative δ^13^C collagen, −10‰ (V-PDB), is higher than what would be expected for a signal from marine primary production^22^. A second alternative explanation is a freshwater tropic web with an anomalous enriched ^13^C source of carbon (DIC) from dissolution or thermic decomposition of carbonates. This geochemical scenario is present in some areas of Italy^34^ in which natural water becomes enriched in CO_2_. This archaeological site was near to the Adige River that runs through Verona. Therefore, water samples from the Adige river and its tributaries were collected and analysed. In addition, ^14^C dating was done on some human remains to detect the presence of “dead carbon”.

The main objective of this study is to demonstrate that these less negative δ^13^C values are related to the consumption of C_4_ plants and refute the improbable hypothesis of a significant marine or anomalous fresh water dietary intake (enriched in ^13^C). At the same time, we set out to quantify the proportion of C_4_ plants in the diet and propose an interpretation about the alimentary habits of Celtic populations of Italy which up to now have been elusive.

## Results

### Isotopic values of the human and animal sample from Verona

**Figure 1:**
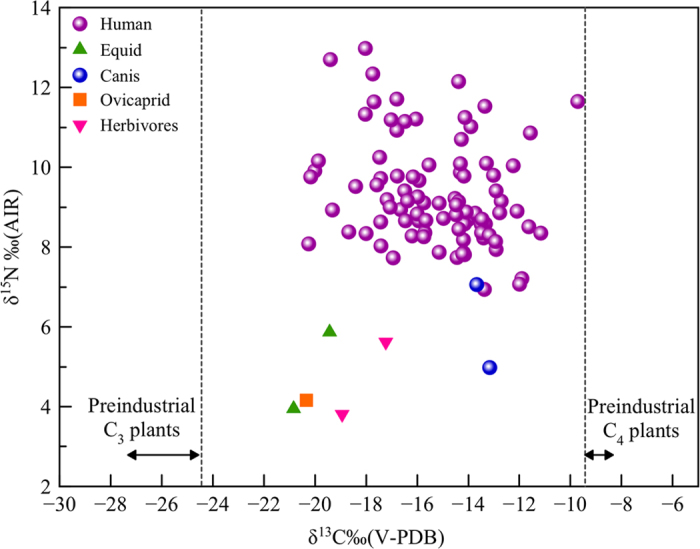
Scatter plot of the data for δ^15^N and δ^13^C analyses of human and animal samples from Verona. The δ^13^C data of human bone collagen range between −20.2 and −9.7‰ (V-PDB), and reveals the significance of C_4_ plant in the diet, with less negative values indicating an almost exclusive diet of C_4_ plants and the more negative values representing a diet lacking C_4_ plants. The range of δ^15^N values (from 6.9 to 12.9‰ AIR) compared with those of animals suggests a mixed terrestrial diet (meat or dairy products and vegetal foodstuffs).

The human δ^13^C values range from −20.2‰ to −9.7‰ (V-PDB) with a mean value of −15.3‰ (±2.2‰) (V-PDB) while δ^15^N values are between +6.9‰ and +12.9‰ (AIR), showing a mean value of +9.4‰ (±1.3‰) (AIR) (Fig. 1; Table 1; Supplementary Table S2). The atomic C/N ratio of the samples falls inside the range of 2.9–3.6, which corresponds to typical values of well-preserved samples, as suggested by DeNiro^35^. Isotopic data from animal bones (Tables 1 and 2) suggests that domesticated herbivores had a diet mainly based on C_3_ plants, although one of these (VRAR-1 with −17.2‰ V-PDB) showed a less negative δ^13^C value that could suggest that this animal partly fed on C_4_ plants. Dogs (n = 2) usually eat men’s leftovers, showing relatively high δ^13^C values (≈ −13‰ V-PDB). One of them also has a relatively low δ^15^N value for an omnivore. This implies a diet poor in animal proteins, supporting the hypothesis of an important influence in their diet of human food scabs, rich in (C_4_) plants.

### Isotopic values of DIC of Adige’s river and Radiocarbon data

The DIC δ^13^C data (Table 3 and Supplementary Figure S1b) of the Adige river ranges between −4.5‰ and −5.0‰ (V-PDB); this, after isotopic fractionation processes of photosynthesis in algae, would result in values ranging from −23.5‰ and −24‰ (V-PDB) and consequently it would imply δ^13^C values in fish tissues very close to these values (≈ −22.5‰ to −23‰ V-PDB). These data would result in a value for human collagen resulting from an exclusively fish diet (an extreme that would be relatively rare), between −20‰ to −22‰ (V-PDB), which is not compatible with the least negative values found in this work. The rest of δ^13^C values of DIC of other tributaries, wells, etc. are even more negative so they also cannot justify the higher δ^13^C values found in collagen of human remains^36^.

On the other hand, the age obtained from ^14^C dating of bone collagen from four individuals (those with less negative δ^13^C values) is consistent with that extrapolated (Table 4) from the preliminary archaeological analysis of the grave goods^36^. This indicates the absence of “dead” carbon (e.g. dissolution of marine carbonate rocks), which would lead to apparently much older ages. Such dissolved inorganic carbon (DIC) have δ^13^C values close to +0‰ or positive values, which would also lead to an aquatic trophic chain with less negative values of δ^13^C, this being the only alternative to the consumption of C_4_ plants. The absence of “dead” carbon in the humans collagen rules out this hypothesis, and thus consequently supports the presence of C_4_ plants in the diet of these individuals.

## Discussion

**Figure 2:**
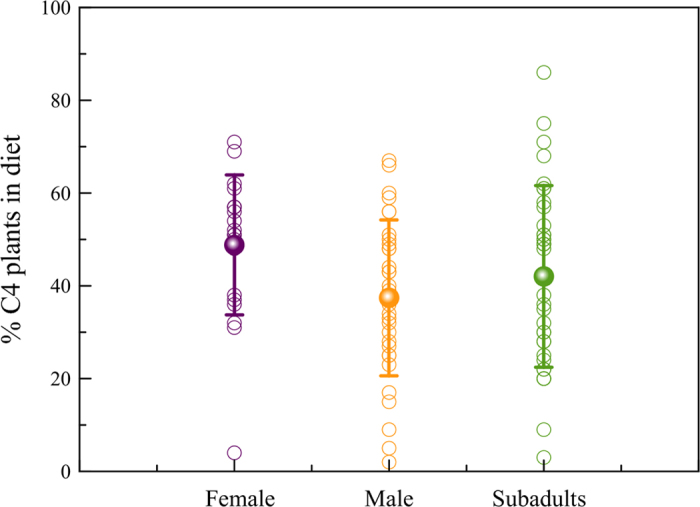
C_4_ plants minimum percentages (%) in the diet of the individuals from Verona. Some individuals show C_4_ plants proportion above 60% in their alimentation, and especially female adults reach significantly higher percentages of C_4_ plants consumption compared to adult males.

**Figure 3:**
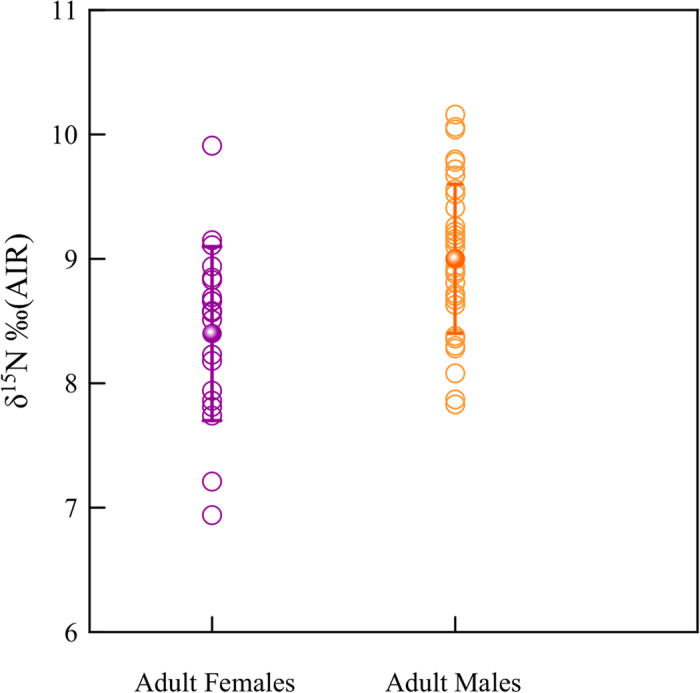
δ^15^N values of adult females and males. Adult males have higher δ^15^N values compared to adult females.

The isotopic results show the important role that C_4_ plants had in the diet of this Celtic population from Verona (Fig. 1). Generally, δ^13^C values of −20‰ (V-PDB) or less negatives would indicate some C_4_ plant contributions. Thus the vast majority of studied individuals (over 90%) seem to include C_4_ plants in a direct way (or indirectly through the consumption of herbivores that fed on them) in their diet. Additionally, adult females (mean value of 48.8 ± 15.1%) show significantly (Mann-Whitney U test = 198.0; p = 0.008) higher consumption of C_4_ plants than adult males (mean of 37.4 ± 16.8%) (Fig. 2). We also found a significant statistical difference in δ^15^N values between sex in the adult group (t = 3.15 p ≤ 0.01): men display higher δ^15^N values compared to women (Fig. 3). Thus, there is a clear differentiation in the diet according to sex with a higher intake of animal protein (meat and meat products) for men, while the diet of women included more cereals and vegetable proteins. It is important to note, that some adults related with high intakes of animal protein (highest values in δ^15^N) have less negative values in δ^13^C (≈−12‰ vs V-PDB, see for example individuals VRSV-12 and VRSV-24 in the Supplementary Table S2), indicating that domestic herbivorous have also consumed large amount of C_4_ plants, although the few herbivorous studied show a consumption of C_3_ plants. However, two dogs show δ^13^C values which are very similar to those of humans (Fig. 1). Especially the younger dog (VRSV-94) presents a typical value of the middle-low animal protein intake of omnivores: δ^15^N = +7.1‰ (AIR). While the δ^13^C value of −13.7‰ (V-PDB) is very close to those of the humans with a C_4_ diet (Table 2). As mentioned before, a possible explanation is that dogs were fed with the impoverished-protein waste from the meals of their owners.

DIC values (Table 3) from local meteoric water show relatively negative δ^13^C values (from −4.5 to −13.3‰ vs V-PDB; i.e. an aquatic diet should be approximately between −23.5 and −32.3‰ vs V-PDB) and ^14^C values of the human samples with less negative δ^13^C_collagen_ values does not indicate the presence of “dead carbon”. Consequently, this excludes a freshwater diet to explain the measured less negative isotopic values of this particular Celtic population and thus supports the hypothesis of a large consumption of C_4_ plants in their diet. Furthermore, δ^15^N values in humans (+9.4‰ AIR) plot in about a one trophic level above terrestrial herbivorous (+4.7‰ AIR)^36^, rule out a marine diet, in agreement with the relative large distance from the coast.

Considering that there are not many known cultivated C_4_ plant species in this time period (3^rd^-1^st^ BC), the hypothesis of millet consumption seems reasonable based on the archaeological record and ancient written source. In fact, the investigated necropolis is located in an area which is still today characterized by a fertile plane (called *Pianura Padana* or Po valley): it offers ideal climatic and environmental conditions for the cultivation of C_4_ plants during the warm seasons (spring-summer). Pliny the Younger (*Epistulae*, IV, 6,1) praised the Po Valley region and wrote “*in regione Transpadana summa abundantia*”^37^. Since the Middle and Late Bronze age (1550-1170 years BC) this plane was characterized by intensive agriculture, pastoralism and the demand of large amounts of wood for building sites. This lead to a heavy deforestation of the whole Po plain and converting it into an artificial steppe devoted to cereal crops^38^. Furthermore, paleoclimate records of lake sediments and speleothems show trends towards a drier climate, or alternating wet and dry climates, between 2500 and 2000 BP^39^,^40^. This might have induced a change in cropping strategies and the consequent introduction of C_4_ plant cultivation (probably millet).

Unfortunately, during the archaeological excavation of Verona, archaeologists have not found seeds (or charcoal) and the ceramic grave goods (vessels and others) are still under study and initially seem not to indicate macroscopic remains of organic waste. Some previous archaeobotanical studies of Neolithic, Bronze and Iron Age sites of the NE of Italy, and particularly of the area of Verona, seem to confirm the presence of *Panicum Miliaceum* and of *Setaria italica* among the carpological remains^41^,^42^. Another comparative paleobotanical study relating to some Iron Age sites located between the Northern and Southern Alps (Eastern Switzerland, Austria and Northern Italy), also describes the presence of millet (*Panicum Miliaceum* L.) and foxtail millet (*Setaria Italica* L. Beauv.) remains^43^. This is also confirmed by the results of the study about carpological remains found at the site of Oppeano (Verona), dated to the second Iron Age (about 6^th^ to 3^rd^ century BC) and located in the same geographical context of the *Seminario Vescovile* necropolis. The majority of the determined cereal remains correspond to millet (*Panicum miliaceum* L.) and foxtail millet (*Echinochloa crus-galli* L. Beauv. and *Setaria italica* L. Beauv)^44^. Thus, the Iron Age seems to be characterized by a greater crops specialization that fits with the assumption of the pursuit of a cereal type that best fits different climates (biomass increase with more efficiency in water use). In this case of the sandy *Pianura Padana* (Verona), the crops of millet were preferred, because it is a plant that adapts very well to poor substrates characterized by water scarcity during summer^44^.

**Figure 4:**
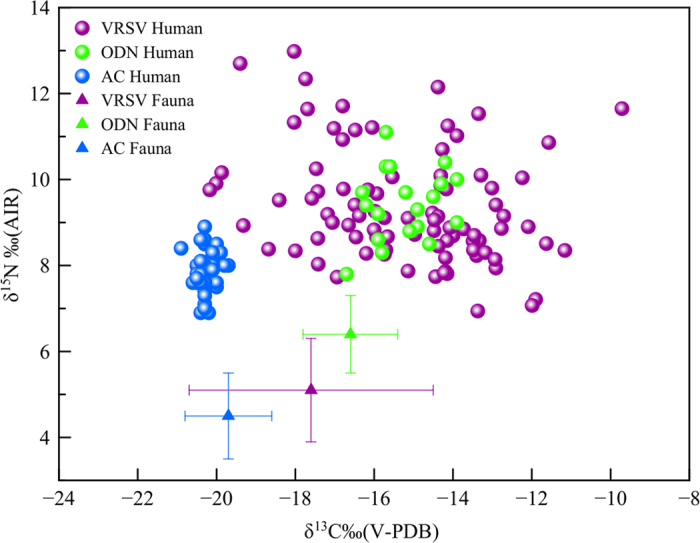
Stable carbon and nitrogen isotope data of human bone collagen from Verona (VRSV) compared with the individual’s values from Olmo di Nogara (ODN)^45^ and Arano di Cellore (AC)^46^. We have also plotted the faunal ranges from the three necropolises. While Verona and Olmo di Nogara (Middle Bronze Age) have very similar isotopic values, confirming a largely C_4_ plants based diet, Arano di Cellore, dated to Early Bronze Age shows a diet based on C_3_ cereal-type plants.

Finally, other isotopic results from bone collagen analysis of some individuals proceeding from the Bronze Age necropolis of Olmo di Nogara, Verona (1600-1200 cal. BC) and of Arano di Cellore, Verona (2040-1890 cal. BC) support the hypothesis of C_4_ plants consumption, and in particular of millet in some areas of the Verona province^45^,^46^(Fig. 4). In conclusion, to these archaeological data we add the testimonies of some ancient authors such as Pliny the Elder (*Naturalis Historiae* XVIII, 83-84) and Columella (*De Re Rustica,* 2, 9, 14–16) who report the use of millet flour for the production of bread and a sort of porridge cooked in water and salt and often accompanied with vegetables and cheese and very rarely with meat. Pliny (XVIII.XXIV) exactly noted: “*millet is used to prepare a very white puls (i.e. similar to present-day polenta). Panic, when ground and freed from bran, and millet as well, makes a porridge which, especially with milk, is not to be despised even in time of plenty*”. Columella (2.9.19), agreed with Pliny and wrote: “*bread is made of millet, and it may be eaten without distaste before it cools*”.

The isotopic data obtained in this study reveal that the proportion of C_4_ plants was substantial (above ≈40%) in the daily diet of this Celtic population. Traces of C_4_ plant consumption are reported in other studies^45^,^46^ from more ancient necropolis of the zone. While Olmo de Nogara (ODN) dated to Middle Bronze Age share very similar isotopic values with Verona (Fig. 4), confirming a preponderant C_4_ plants based diet, Arano di Cellore (AC), dated to Early Bronze Age, shows a diet based on the consumption of C_3_ cereal-type plants. Hence, the data from AC and ODN firmly place the shift in C_4_ crop use in the region at a transition period between the late phases of the Early Bronze Age and the beginning of the Middle Bronze Age^46^, consequently our data show that successively, in pre-Roman times, the diet of most habitants of the zone was almost exclusively based on C_4_ plants. This, indirectly indicates that this Celtic population had mastered agriculture techniques to such an extent that they could live off the harvest of their cultivated crops the year round.

## Methods

A sample of 90 human ribs, consisting of both sexes and different ages (Supplementary Information Table S1), and 7 animal bones, was selected for analysis of bone collagen. The animal bone sample is composed of 5 herbivorous species (2 horses, 1 goat/sheep and 2 cows) and 2 omnivorous species (2 dogs). The extraction of collagen is performed using the protocol described by Bocherens *et al*.^47^,^48^ and following the routine procedures of the Stable Isotope Biogeochemistry Laboratory of the Andalusian Institute of Earth Sciences (CSIC, Granada, Spain). We also determined the current DIC values of the Adige’s river (and of some of its tributaries) and dated with ^14^C the collagen samples of the less negative human values (a source of “dead” inorganic carbon would show abnormally high ages).

The δ^13^C mean values of present day C_3_ and C_4_ plants are respectively −26‰ and −11‰ (V-PDB)^49^,^50^, but before the industrial revolution the isotopic composition of atmospheric CO_2_ was 1.5‰ less negative^51^. Hence, to make a more precise estimate we considered that these two carbon sources would respectively correspond to values of −24.5‰ and −9.5‰ (V-PDB) (see Supplementary Information for detailed descriptions about the different methodologies applied).

## Additional Information

**How to cite this article**: Laffranchi, Z. *et al*. Stable C & N isotopes in 2100 Year-B.P. human bone collagen indicate rare dietary dominance of C4 plants in NE-Italy. *Sci. Rep.* **6**, 38817; doi: 10.1038/srep38817 (2016).

**Publisher's note:** Springer Nature remains neutral with regard to jurisdictional claims in published maps and institutional affiliations.

## Supplementary Material

Supplementary Information

## Figures and Tables

**Table 1:** Summary table with Number of samples (N), Minimum (Min), Maximum (Max), Mean (M) and standard deviation (SD) of the isotopic values (‰) of human and animal remains.

	δ^15^N‰ (AIR)					δ^13^C‰(V-PDB)				
	N	Min	Max	M	SD	N	Min	Max	M	SD
Humans	90	6.9	12.9	9.4	1.3	89	−20.2	−9.7	−15.3	2.2
Herbivores	5	3.8	5.9	4.7	0.9	5	−20.8	−17.2	−19.3	1.4
Omnivores	2	4.9	7.1	6.0	1.6	2	−13.7	-13.2	−13.4	0.3

**Table 2:** Isotopic values (δ^15^N and δ^13^C) of the animal samples with the estimated percentage of C_4_ plants in their diet. %PP-C_4_: minimum percentages of diet based on C_4_ primary production.

Sample	Species	δ^15^N ‰AIR	δ^13^C ‰V-PDB	Bone	C/N	%PP- C_4_
VRSV-92	Equid	5.9	−19.4	long bone	2.9	9
VRSV-93	Canis	4.9	−13.2	cranium	2.9	59
VRSV-94	Canis	7.1	−13.7	cranium	3.3	55
VRSV-95	Equid	3.9	−20.8	long bone	3.0	0
VRSV-96	Ovicaprid	4.2	−20.3	tibia	3.2	1
VRAR-1	Herbivore	5.6	−17.2	long bone	3.2	26
VRAR-2	Herbivore	3.8	−18.9	vertebra	3.2	12

**Table 3:** DIC (dissolved inorganic carbon) isotopic composition of the water of the zone.

Sample	ID water	Localization	δ^13^C_DIC_ ‰ (V-PDB)
1	Alpone creek	Vestenanova (VR)	−9.7
2	Progno creek	Giazza (VR)	−4.6
3	Tana delle sponde cave	Velo Veronese (VR)	−4.9
4	Bassanella source	Soave (VR)	−13.3
5	Well’s water (108 m)	Gambellara (VI)	−10
6	Adige river	Bolzano (BZ)	−5
7	Avesa creek	Avesa (VR)	−9.2
8	Adige river	Chievo dam (VR)	−4.5
9	Fontana del Ferro source	Verona	−11.7

**Table 4:** ^14^C dating of the less negative δ^13^C human collagen samples.

Sample	δ^13^C ‰ V-PDB	YEARS B.P.	YEARS B.C. (2σ)	LAB ID
VRSV-12	−12.7	2080 ± 32	193–37	CAN-2880
VRSV-16	−11.9	2164 ± 33	261–110	CNA-2881
VRSV-17	−16	2113 ± 32	204–47	CNA-2882
VRSV-24	−12.2	2149 ± 32	234–89	CNA-2883
