# Supplementary material for: Stable C & N isotopes in 2100 Year-B.P. human bone collagen indicate rare dietary dominance of C4 plants in NE-Italy
Source: Sci Rep. 2016 Dec 9;6:38817. doi: 10.1038/srep38817 (PMC5146934; doi:10.1038/srep38817)
Supplement: Supplementary Information [file srep38817-s1.pdf]

Supplementary Information for:

Stable C & N isotopes in 2100 Year-B.P. human bone collagen indicate rare dietary dominance of C4 plants in NE-Italy.

Zita Laffranchi[1*], Antonio Delgado Huertas[2], Sylvia A. Jiménez Brobeil[1], Arsenio Granados Torres[2], Jose A. Riquelme Cantal[3].

[1]Department of Legal Medicine, Toxicology and Physical Anthropology, Medicine Faculty, University of Granada (UGR), Av. de la Investigación 11, 18016, Granada, Spain.

[2] Biogeochemical of Stable Isotopes Laboratory, Andalusian Institute of Earth Sciences (IACT-CSIC-UGR), Av. de las Palmeras 4, 18100, Armilla, Granada, Spain.

[3]Departament of Geography and Territorial Sciences, Area of Prehistory, University of Cordoba, Plaza Cardenal Salazar 3, 14971, Cordoba, Spain.

Archaeological context

During the construction of an underground garage in the main courtyard of the Bishop's Seminary (*Seminario Vescovile*) at Verona (Italy) archaeologists discovered between 2005 and 2010 a large Pre-Roman necropolis (3rd to 1st century BC) from a Roman-influenced Celtic tribe (*Cenomani* Gauls). The Bishop's Seminary is located in the eastern Veronetta district of Verona (Supplementary Figs. S1a and S1b) outside the Republican city walls between the left bank of the river Adige and the first hills. The site is approximately 250 m to the west of the current river bank and about 67 m to the east of the ancient Roman road to Postumia (Thompson and Bersani, Unpublished report).

The necropolis contains approximately 163 simple burial graves with a minimum of 174 skeletons in a good state of preservation. The majority of individuals are non-adults (108 individuals), and there are 66 adults. The burials are currently under archaeological study and results have not yet been published. The necropolis was initially attributed to the 2nd century BC, based on the type of funeral grave goods observed in a preliminary study of the metal materials. However, some preliminary absolute dating from bone collagen analysis in some skeletons suggests its utilization from the 3rd century BC[33].

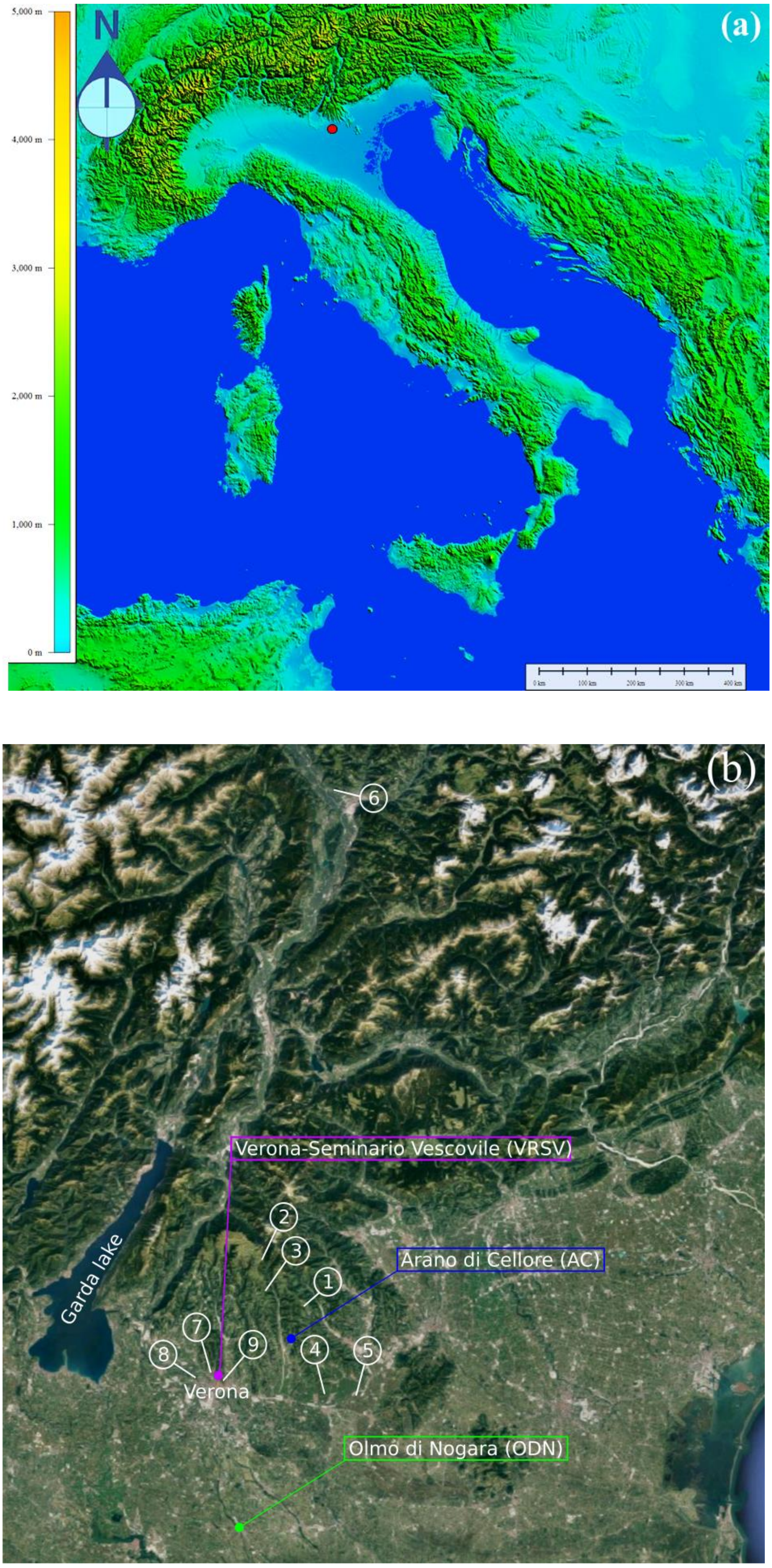
5,000 m
4,000 m
3,000 m
2,000 m
1,000 m
0 m
N
(a)
0 km
100 km
200 km
300 km
400 km
(b)
6
Verona-Seminario Vescovile (VRSV)
2
3
1
Arano di Cellore (AC)
Garda lake
7
8
9
4
5
Verona
Olmo di Nogara (ODN)

Supplementary Figure S1. (a) Geographical localization of the city of Verona, Italy (the map was generated with Global Mapper 16.1, URL: http://www.bluemarblegeo.com/products/global-mapper.php). **(b)** Localization of the necropolises of Seminario Vescovile (VRSV), Olmo di Nogara (ODN) and Arano di Cellore (AC). The points where water samples were collected for DIC's analysis are also indicated. The numbers (1-9) refer to those presented in Table 2 (the map was created by Google Earth, Data SIO, NOAA, U.S. Navy, NGA, GEBCO, Image Landsat, URL: https://www.google.it/intl/it/earth/ and modified with Inkscape 0.91, URL: https://inkscape.org/en/).

The individuals of this necropolis are generally laid out in supine position in single graves, whereas the typical funerary rituals in other La Tène cemeteries include not only inhumations but also cremations, which were more frequent. The burials are all simple earth-graves. Grave goods, which are documented in most but not all of the burials, include animal bones, ceramics, decorations, and weapons, although the latter are rare. There are two individuals buried with complete skeletons of animals: the first is VRSV-68 a neonate buried with a dog (VRSV-93) (Supplementary Fig. S2) while the second is a mature female VRSV-21 (Supplementary Fig. S3), buried with a complete skeleton of a horse (VRSV-92) and a partial skull of a dog (VRSV-94)[36]. The chronology of the burials is associated with a period of cultural and political transition in which Celtic populations (late Iron Age groups) lived alongside the first Roman communities in the region[33].

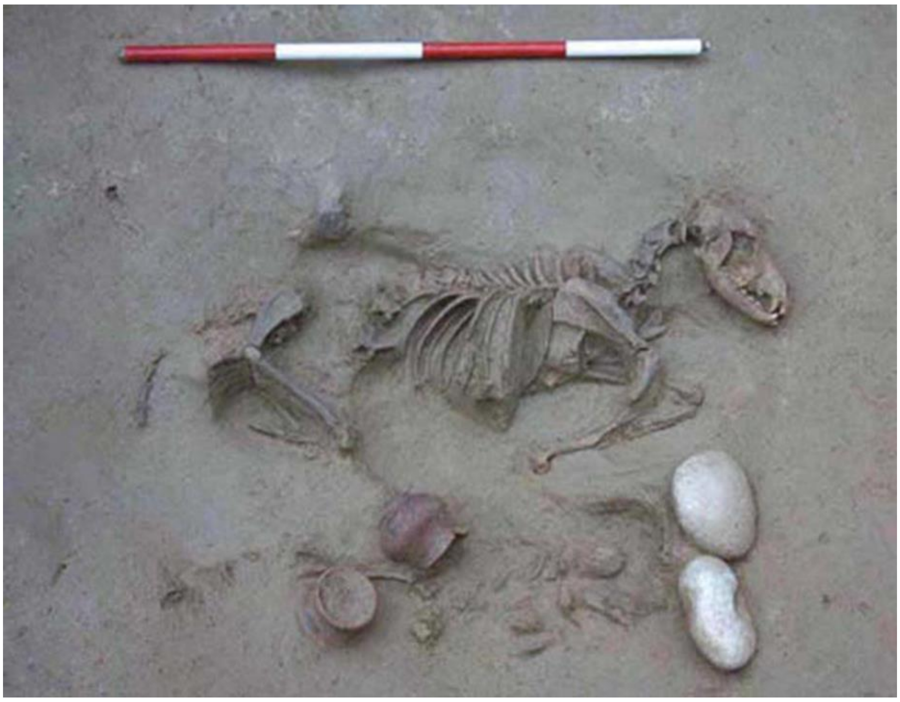

Supplementary Figure S2. Tomb of a neonate (VRSV-68) buried with a mature dog (VRSV-93) and some ceramic vessels (Photo: Simon Thompson, with his kind permission).

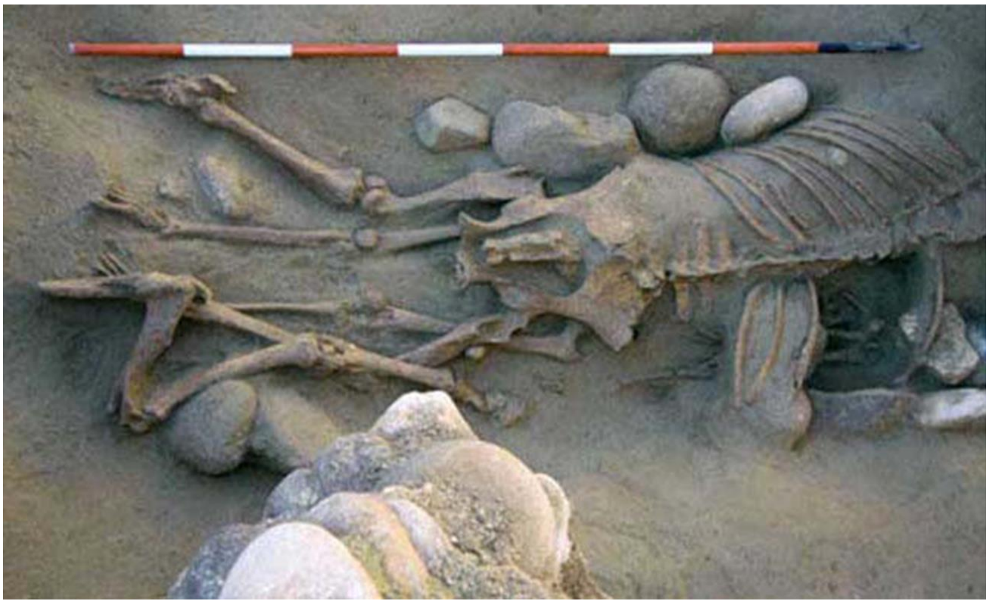

Supplementary Figure S3. A detail view of the mature female VRSV-21 lying under the skeleton of the horse VRSV-92 (Photo: Simon Thompson, with his kind permission).

Methods

Anthropological analysis

The human selected sample is composed of 90 individuals: 36 subadults and 54 adults (Supplementary Table S1). The adults were aged with the following methods: morphological changes of the pubic symphysis and on the auricular surface of the ilium, morphological changes at the sternal rib ends and cranial suture closure[52]. The age of the subadults was estimated with the following criteria: development and eruption of the deciduous and permanent dentition, development of the temporal and occipital bones, development and fusion of the epiphyses and maximum length of long bones diaphysis[53,54].

Supplementary Table S1. Sex and age distribution of the Seminario Vescovile' skeletal sample.

AGE (years)	SEX			
	**Male**	**Female**	**Unknown**	**Total**
Subadult (< 21)	-	-	36	36
Adult (21-40)	19	9	-	28
Mature (41-60)	14	12	-	26
Total	46	36	8	90

Stable isotopes

The extraction of collagen is based on the protocol described by Bocherens et al. (1991, 1997[47,48]). In a first stage, collagen is extracted from the bone by a chemical procedure that ensures the removal of other organic compounds that might contaminate

the sample. Rib samples were initially cleaned by scratching and then they were powdered to a particle size of less than 0.7 mm. About 300 mg of bone powder is decalcified in 1 M HCl for 20 minutes at room temperature, eliminating phosphates, fulvic acids and other soluble acids and filtered through a MF-Millipore 5 μm filter. The insoluble residue is plunged into 0.125 M of NaOH for 20 hours at room temperature. After rinsing with Milli-Q water, the neutralized sample is filtered again (5 μm) to remove humic acids and most lipids; this residue is plunged into 10^{-2} M HCl (pH 2) solution in closed pyrex tubes, at 100° for 17 hours to solubilize the collagen. After centrifugation of the tubes at 6300 rpm for about 10 minutes, the supernatant (containing solubilized collagen) is lyophilized and analyzed for its isotopic composition. About 1 mg of collagen was weighed into a tin capsule. Samples were analysed for the isotopic composition of nitrogen and carbon by means of a Carlo Elba NC1500 (Milan, Italy) elemental analyser on line with a Delta Plus XL (ThermoQuest, Bremen, Germany) mass spectrometer (EA-IRMS).

DIC samples were prepared in order to liberate CO_2 as follows: an aliquot of sample was injected into 12-ml vials pre-filled with helium and 5 drops of 65% phosphoric acid and shaken in a Vortex agitator for 30 seconds. The vials were then left at room temperature for between 15 and 36 hours to obtain a state of equilibrium[55]. The CO_2 was separated from other residual gases by chromatography using a helium carrier gas in a Gas Bench (Thermo Finnigan, Bremen, Germany) system interfaced with a mass spectrometer[56]. For DIC samples, isotopic ratios were measured using Delta XP mass spectrometer (IRMS).

The stable composition is reported as δ values per mil:

$$\delta = (R_{sample}/R_{standard} - 1) * 1000$$

where R= $^{13}C/^{12}C$ for $\delta^{13}C$ values and R= $^{15}N/^{14}N$ for $\delta^{15}N$.

Commercial CO_2 and N_2 were used as the internal standard for the carbon and nitrogen isotopic analyses. For carbon 22 internal standard (organic and inorganic material) ranging between -49.44 ‰ to +28.59 ‰ (V-PDB), contrasted with the IAEA international references NBS-28, NBS-29, NBS-20 (carbonates) and NBS-22, IAEA-CH-7, IAEA-CH-6 (organic material), are used in relation to the isotopic range of samples to be analyzed. For this study 2 internal standard of -30.63‰ and -11.65 ‰ (V-PDB) have been used. For nitrogen, 9 internal standard (organic and inorganic material) ranging between -1.94 ‰ to +16.01 ‰ (AIR), contrasted with the IAEA international references IAEA-N-1, IAEA-N-2, NO-3, USGS32, USGS34 and USGS35. In this study 2 internal standard of -1.02‰ and +16.01 ‰ (AIR) have been used. For DIC 3 internals standards of Na_2CO_3 solution have been used (DIC-A, DIC-B and DIC-T), each with a different isotopic composition (-4.9‰, -9.50‰, +28.59‰ vs V-PDB).

All internal solid standards, including three liquid standards of DIC (A, B and T) of about 15 L (preserved at room temperature by poisoning with mercuric chloride), of which an aliquot was precipitated as barium carbonate, were measured with an Elemental Analyzer online with a Delta Plus XL mass spectrometer (IRMS). Additionally, the carbonates were also measured with a Gas Bench (Thermo Finnigan, Bremen, Germany) system interfaced with a mass spectrometer (Delta XP). Precision calculated, after correction of the mass spectrometer daily drift, from standards systematically interspersed in analytical batches was better than ± 0.1‰ for $\delta^{15}N$ and $\delta^{13}C$ in collagen and DIC. The standard for reporting carbon measurements is V-PDB (Vienna-PDB)[56] and for nitrogen measurements is atmospheric nitrogen (AIR).

^{14}*C dating*

The preparation protocol including ultrafiltration, is a modified version of the Longin (1971)[57] procedure to obtain the collagen[57]. Basically, HCl 10^{-2} M is added and pH adjusted to 2.0–2.5. It is then kept at 58ºC for two days. Ezee-filters™ (Elkay Laboratory Products, 9 ml, 60–90 lm) are used to eliminate undissolved material. The dissolved collagen is transferred to previously cleaned ultrafilters (Amicon Ultra-4, Millipore, 30 KDa), and centrifuged to eliminate the short collagen molecules. The final product obtained after ultrafiltration is freeze-dried[58]. Samples for ^{14}C dating were analyzed by AMS (Accelerator Mass Spectrometry) at the *Centro Nacional de Aceleradores* (CNA, Sevilla, Spain) with a 1 MV multielemental AMS Micadas, giving a 200 kV radiocarbon dating[59].

Theoretical Calculus:

For the theoretical calculus, starting from a conservative model to obtain minimum percentages of diet based on C_4 primary production (**% PP-C_4**), we considered that collagen values of a human whose diet is based on C_3 plants show more negative δ^{13}C values. The most negatives in this series are close to -20.5‰ (V-PDB), though we observed an accumulated fractionation to an omnivore diet of 4‰ but they were probably affected by a low consumption of C_4 plants of primary production in the trophic web. However, for a more direct use, as in the case of C_4 cereals, we chose a medium value between +1‰ (as suggested in literature[32] for the carbon at each trophic level) and +5‰ thus we considered as quite probable a fractionation of +2.5‰ for calculations. Specifically, for the calculation of C_4 plants minimum percentages in the

diet we propose the following equation that should be considered as a simple approximation with an error near to 5%:

% PP-C_4 = [100/ (theoretical collagen C_4-C_3)]*(δ^{13}C sample – (-20.5))

Where % PP-C_4 is the proportion of primary production based on C_4 plants in the diet. Collagen from C_3 plants consumption would have a hypothetical value of -20.5‰ (V-PDB) and C_4 plants of -8 ‰ (V-PDB).

For example, we applied this equation to the sample VRSV-11 with a δ^{13}C value of -14 ‰ (V-PDB):

% PP-C_4 = [100/(-8) – (-20,5)]*(-14) - (-20,5)) = [100/12,5]*(6,5) = 52 %.

Therefore, the percentage of C_4 plants in the diet of the individual VRSV-1 is of 52%.

Supplementary Table S2. Isotopic values (δ^{15}N and δ^{13}C) of the human samples with the estimated percentage of C_4 plants in their diet. F: female; M: male.

Sample	Sex	Age	δ^{15}N‰ AIR	δ^{13}C‰ V-PDB	C/N	% PP-C_4*
VRSV-1	F	Mature	6.9	-13.4	3.4	57
VRSV-2	F	Adult	8.9	-16.6	3.3	31
VRSV-3	F	Mature	7.8	-14.1	3.1	51
VRSV-4	F	Mature	8.7	-15.9	3.0	37
VRSV-5	F	Mature	9.1	-15.7	3.1	38
VRSV-6	F	Adult	7.9	-12.9	3.1	61
VRSV-7	F	Senil	8.2	-13.4	3.1	57
VRSV-8	F	Mature	8.2	-14.2	3.3	51
VRSV-9	F	Adult	8.7	-16.5	3.2	32
VRSV-10	F	Mature	8.6	-13.3	2.9	57

VRSV-11	F	Adult	8.7	-14	2.9	52
VRSV-12	F	Adult	9.1	-12.7	3.1	62
VRSV-13	F	Adult	7.7	-14.4	3.0	48
VRSV-14	F	Mature	7.9	-14.2	3.0	50
VRSV-15	F	Mature	8.6	-14.1	3.1	51
VRSV-16	F	Mature	7.2	-11.9	3.2	69
VRSV-17	F	Mature	8.8	-16	3.1	36
VRSV-18	F	Adult	8.5	-11.6	3.4	71
VRSV-19	F	Adult	9.9	-20	3.3	4
VRSV-20	F	Adult	8.8	-13.7	3.5	54
VRSV-21	F	Mature	8.6	-13.5	3.3	56
VRSV-22	M	Adult	9.2	-17.2	3.1	27
VRSV-23	M	Adult	9.8	-13	3.1	60
VRSV-24	M	Adult	10	-12.2	3.2	66
VRSV-25	M	Mature	8.1	-20.2	3.1	2
VRSV-26	M	Adult	8.4	-15.7	3.1	38
VRSV-27	M	Adult	9.4	-16.5	3.1	32
VRSV-28	M	Mature	8.9	-19.3	3.0	9
VRSV-29	M	Mature	8.7	-13.5	3.1	56
VRSV-30	M	Adult	8.6	-17.4	2.9	25
VRSV-31	M	Adult	9	-17.1	2.9	28
VRSV-32	M	Adult	8.9	-14.1	3.1	51
VRSV-33	M	Mature	8.4	-13.5	3.1	56
VRSV-34	M	Mature	8.3	-16.2	3.0	34
VRSV-35	M	Adult	8.4	-18.7	3.4	15
VRSV-36	M	Adult	8.7	-15.7	3.6	39
VRSV-37	M	Mature	7.9	-15.1	2.9	43
VRSV-38	M	Adult	8.7	-14.9	3.1	44
VRSV-39	M	Mature	8.8	-14.5	2.9	48
VRSV-40	M	Mature	9.2	-14.5	2.9	48

VRSV-41	M	Mature	7.8	-14.2	3.0	50
VRSV-42	M	Mature	9.8	-16.8	2.9	30
VRSV-43	M	Adult	9.3	-15.9	3.2	36
VRSV-44	M	Adult	10.1	-15.5	3.2	40
VRSV-45	M	Adult	9.7	-17.4	3.2	25
VRSV-46	M	Mature	9.1	-14.4	3.2	49
VRSV-47	M	Adult	9.1	-15.1	3.2	43
VRSV-48	M	Adult	8.9	-12.1	3.2	67
VRSV-49	M	Adult	8.3	-13.2	3.2	59
VRSV-50	M	Mature	9.2	-16.4	3.1	33
VRSV-51	M	Mature	9.5	-18.4	3.1	17
VRSV-52	M	Adult	9.7	-15.9	3.1	37
VRSV-53	M	Adult	9.6	-17.6	3.1	23
VRSV-54	-	Subadult	7.7	-16.9	3.1	28
VRSV-55	-	Subadult	11.2	-16	3.2	36
VRSV-56	-	Subadult	12.9	-18	3.1	20
VRSV-57	-	Subadult	11.1	-16.5	3.1	32
VRSV-58	-	Subadult	11.5	-13.3	3.1	57
VRSV-59	-	Subadult	8.3	-18	3.0	20
VRSV-60	-	Subadult	9.9	-14.3	3.2	49
VRSV-61	-	Subadult	8.9	-12.8	2.9	62
VRSV-62	-	Subadult	9.8	-20.2	3.2	3
VRSV-63	-	Subadult	11.6	-17.7	3.2	22
VRSV-64	-	Subadult	9.4	-12.9	2.9	61
VRSV-65	-	Subadult	10.9	-11.6	2.9	71
VRSV-66	-	Subadult	8	-17.4	2.9	25
VRSV-67	-	Subadult	10.9	-16.8	3.2	30
VRSV-68	-	Subadult	11	-13.9	2.9	53
VRSV-69	-	Subadult	8.3	-11.2	3.2	75
VRSV-70	-	Subadult	10.2	-	-	-

VRSV-71	-	Subadult	10.7	-14.3	2.9	50
VRSV-72	-	Subadult	11.2	-14.1	3.2	51
VRSV-74	-	Subadult	7.1	-11.9	3.2	68
VRSV-75	-	Subadult	9.8	-14.2	3.3	51
VRSV-76	-	Subadult	10.1	-13.3	3.2	58
VRSV-77	-	Subadult	10.2	-17.5	3.1	24
VRSV-78	M	Mature	10.2	-19.9	2.9	5
VRSV-79	-	Subadult	9.1	-14.5	2.9	48
VRSV-80	-	Subadult	11.3	-18	3.2	20
VRSV-81	-	Subadult	11.2	-17	3.3	28
VRSV-82	-	Subadult	10.1	-14.3	3.1	50
VRSV-83	-	Subadult	11.6	-9.7	3.3	86
VRSV-84	-	Subadult	12.1	-14.8	3.2	49
VRSV-85	-	Subadult	12.3	-17.7	3.1	22
VRSV-86	-	Subadult	11.7	-16.8	3.1	30
VRSV-87	-	Subadult	8.3	-15.7	3.2	38
VRSV-88	-	Subadult	12.7	-19.4	3.3	9
VRSV-89	-	Subadult	8.1	-12.9	3.3	61
VRSV-90	-	Subadult	9.8	-16.2	3.3	35
VRSV-91	-	Subadult	8.4	-14.4	3.2	49

* minimum percentages of diet based on C_4 primary production (**% PP-C_4**).

References

52. Byers, S.N. *Introduction to Forensic Anthropology.* (Pearson/Allyn and Bacon, Boston, 2005).

53. Scheuer, L. & Black, S. *Developmental Juvenile osteology* (Academic Press, London, 2000).

54. Schaefer, M., Black, S. & Scheuer, L. *Juvenile Osteology. A laboratory and field manual* (Academic Press, London, 2009).

55. Salata, G.G., Roelke, L.A. & Cifuentes, L.A. A rapid and precise method for measuring stable carbon isotope ratios of dissolved inorganic carbon. *Mar Chem* **69**, 153–161 (2000).

56. Van Geldern, R. *et al.* Stable carbon isotope analysis of dissolved inorganic carbon (DIC) and dissolved organic carbon (DOC) in natural waters - Results from a worldwide proficiency test. *Rapid Commun Mass Spectrom* **27**, 2099-2107 (2013).

57. Longin, R. New method of collagen extraction for radiocarbon dating. *Nature* **230** (5291) 241–242 (1971).

58. Brock, F., Ramsey, C.B. & Higham, T. Quality assurance of ultrafiltered bone dating. *Radiocarbon* **49 (2),** 187–192 (2007).

59. Santos Arévalo, F.J., Gómez Martínez, I., Agulló García, L., Reina Maldonado, M. T. & García León, M. ^{14}C determination in different bio-based products. Nuclear Instruments and Methods in Physics Research Section B: *Beam Interactions with Materials and Atoms* **361,** 354-357 (2015).
